# Optimization of Control Strategies for Non-Domiciliated *Triatoma dimidiata*, Chagas Disease Vector in the Yucatán Peninsula, Mexico

**DOI:** 10.1371/journal.pntd.0000416

**Published:** 2009-04-14

**Authors:** Corentin Barbu, Eric Dumonteil, Sébastien Gourbière

**Affiliations:** 1 UMR 5244 CNRS-EPHE-UPVD, Laboratoire de Biologie et d'Ecologie Tropicale et Méditerranéenne, Université de Perpignan Via Domitia, Perpignan, France; 2 Laboratorio de Parasitología, Centro de Investigaciones Regionales “Dr. Hideyo Noguchi”, Universidad Autónoma de Yucatán, Mérida, Yucatán, Mexico; 3 Department of Tropical Medicine, School of Public Health and Tropical Medicine, Tulane University, New Orleans, Louisiana, United States of America; Universidad de Buenos Aires, Argentina

## Abstract

**Background:**

Chagas disease is the most important vector-borne disease in Latin America. Regional initiatives based on residual insecticide spraying have successfully controlled domiciliated vectors in many regions. Non-domiciliated vectors remain responsible for a significant transmission risk, and their control is now a key challenge for disease control.

**Methodology/Principal Findings:**

A mathematical model was developed to predict the temporal variations in abundance of non-domiciliated vectors inside houses. Demographic parameters were estimated by fitting the model to two years of field data from the Yucatan peninsula, Mexico. The predictive value of the model was tested on an independent data set before simulations examined the efficacy of control strategies based on residual insecticide spraying, insect screens, and bednets. The model accurately fitted and predicted field data in the absence and presence of insecticide spraying. Pyrethroid spraying was found effective when 50 mg/m^2^ were applied yearly within a two-month period matching the immigration season. The >80% reduction in bug abundance was not improved by larger doses or more frequent interventions, and it decreased drastically for different timing and lower frequencies of intervention. Alternatively, the use of insect screens consistently reduced bug abundance proportionally to the reduction of the vector immigration rate.

**Conclusion/Significance:**

Control of non-domiciliated vectors can hardly be achieved by insecticide spraying, because it would require yearly application and an accurate understanding of the temporal pattern of immigration. Insect screens appear to offer an effective and sustainable alternative, which may be part of multi-disease interventions for the integrated control of neglected vector-borne diseases.

## Author Summary

Chagas disease is the most important vector-borne disease in Latin America. Residual insecticide spraying has been used successfully for the elimination of domestic vectors in many regions. However, some vectors of non-domestic origin are able to invade houses, and they are now a key challenge for further disease control. We developed a mathematical model to predict the temporal variations in abundance of non-domiciliated vectors inside houses, based on triatomine demographic parameters. The reliability of the predictions was demonstrated by comparing these with different sets of insect collection data from the Yucatan peninsula, Mexico. We then simulated vector control strategies based on insecticide spraying, insect, screens and bednets to evaluate their efficacy at reducing triatomine abundance in the houses. An optimum reduction in bug abundance by at least 80% could be obtained by insecticide application only when doses of at least 50 mg/m^2^ were applied every year within a two-month period matching the house invasion season by bugs. Alternatively, the use of insect screens consistently reduced bug abundance in the houses and offers a sustainable alternative. Such screens may be part of novel interventions for the integrated control of various vector-borne diseases.

## Introduction

Chagas disease is a major vector-borne parasitic disease in Latin America, with 9.8 to 11 million infected people, 60 million at risk of infection [1],[2] and a disease burden of over 800,000 DALYs [3]. International travel and immigration are also turning it into a global disease [4]. It is caused by the protozoan parasite *Trypanosoma cruzi*, which is transmitted to humans primarily by triatomine vectors. Due to its importance in public health, vector control strategies have been widely implemented through several regional initiatives in the Americas. These interventions are based on the elimination of domiciliated triatomine vectors by residual insecticide spraying and/or housing improvement, and have resulted in a large reduction in house infestation by triatomines (particularly *Triatoma infestans*), and a corresponding reduction in Chagas disease transmission to humans [1],[2],[5].

However, it has become increasingly clear that several triatomine species do not establish permanent domestic colonies, but can occasionally infest domestic habitats by immigration from peridomestic and/or sylvatic habitats. These species include *Rhodnius prolixus* in Venezuela [6], *Triatoma brasiliensis* and *Triatoma pseudomaculata* in Brazil [7], *Triatoma mexicana* in central Mexico [8], or *Triatoma dimidiata* in the Yucatan peninsula, Mexico and Belize [9],[10].

Extensive field collections of *T. dimidiata* in both rural and urban areas of the Yucatan peninsula revealed a very clear and reproducible seasonal pattern of transient house infestation by predominantly adult triatomines during April-July, associated with a very limited colonization of domiciles [9], [11]–[14]. These data suggested a seasonal dispersal of adult triatomines from nearby peridomestic and/or sylvatic sites, which was confirmed by the analysis of population stage structure [9] and population genetics studies [15]. Mathematical modelling further revealed that dispersal was the dominant parameter involved in this infestation process, while demography was of secondary importance [16],[17]. Finally, analysis of blood-feeding and fecundity of natural populations suggested that foraging for better host-feeding sources may contribute to the seasonal dispersal of *T. dimidiata* [18], and while nutritional status and fecundity tended to improve in the houses, these remained largely suboptimal and may thus contribute to ineffective colonization [18]. Accordingly, *T. dimidiata* populations in the Yucatan peninsula behave as typical source-sink dynamical systems [19],[20], with outdoor habitats as sources and houses as sinks [16]. Another important specificity of these populations is the very low bug abundance observed, which suggests that density dependent process may be of little relevance in the dynamics of the sink habitats [16]. Importantly, variations in this infestation pattern may occur elsewhere as *T. dimidiata* presents extensive ecological, behavioral and genetic diversity [21]–[23].

The control of house infestation by such non-domiciliated triatomine vectors is identified as a major problem and one of the new challenges for Chagas disease control since conventional spraying control strategies may be of limited efficacy in these conditions [2], [24]–[26]. Insecticide spraying has a rather short-lived effect on house infestation in the case of recurring infestation by immigrating peridomestic and/or sylvatic bugs, as we observed in a previous field study on *T. dimidiata* vector control in the Yucatan peninsula [27]. It is thus of key importance to improve and optimize the efficacy of current insecticide spraying strategies to cope with (re)infestation by non-domiciliated vectors and to investigate the potential of alternative strategies such as insect screens or bednets [26],[28],[29]. This can be achieved by empirical field trials [30],[31], but this costly approach is limited in the number of control strategies that can be evaluated and the follow-up time required. Alternatively, the use of mathematical modelling has proven to be a very efficient approach to explore control strategies in a variety of contexts and diseases [32]–[35]. Although some modelling studies have investigated vector population dynamics [16],[17],[32],[36] and Chagas disease transmission [37], very few have attempted to optimize control strategies [32] and none focused on non-domiciliated vectors, most likely because of the lack of estimates of the required population parameters in this situation [24],[26].

In the present contribution, we use a combination of field and modelling studies to evaluate the efficacy of several strategies for the control of seasonal infestation by non-domiciliated triatomine populations. We took advantage of one of the best documented case of non-domiciliated triatomine vector; the populations of *T. dimidiata* in the Yucatan peninsula, Mexico. Our modelling shows that the control of non-domiciliated vectors can hardly be achieved by insecticide spraying, but that insect screens may offer an effective and sustainable alternative.

## Methods

### General overview

We aimed to construct a model able 1) to reproduce and predict the temporal variations of vector abundance in the absence of control, and 2) to account for various control strategies. We expanded a previous population dynamics model [16] to include mathematical descriptions of different control strategies such as insecticide spraying, insect screens, and bednets, for their evaluation. The model predicts the temporal variations in vector abundance in one house as a function of survival and fecundity of triatomines inside the house, the immigration of bugs from peridomestic or sylvatic habitats, and the effect of the above control strategies on those parameters. Estimates of the parameters in the absence of control intervention were obtained by fitting the model to a first set of field data corresponding to the observed variations in the average vector abundance inside houses of two villages where no control actions were applied. The predictive value of the model was then tested on a second independent data set, corresponding to the observed variations in vector abundance inside houses of three other villages with no control interventions. This parametrized model, combined with the description of the effect of insecticide on vector survival and fecundity, was then fitted to a third data set from a field control trial to estimate the half-life of the insecticide. We then used the model to explore the efficacy of varying the timing of insecticide application within the year, the frequency of spraying, and the dose of insecticide used. Similarly, we evaluated the effect of insect screens and bednets by performing a complete sensitivity analysis of their possible effects. The efficacy of any given strategy was evaluated as the percent reduction in the abundance of vectors, in comparison with the expected abundance in the absence of control interventions as evaluated from the model. Finally, we performed a sensitivity analysis of the effect of the number of immigrant bugs, the domestic demography of the vector, the half-life and the lethal effect of the insecticide on the efficacy of the various interventions.

### Field trials

Data on the dynamics of house infestation by triatomines in the absence of vector control interventions were collected over 3 years of field studies, from October 1999 to December 2001 and from January to December 2003 [9],[11],[13]. Triatomines were collected by a standardized methodology based on community participation in 5 villages from Northern Yucatan, Mexico (Dzidzilche, Tetiz, Eknakan, Suma and Izamal). Participating families provided oral consent prior to their participation, as written consent was waived because the study involved no procedures for which written consent is normally required outside of the research context. Consent was logged in field notebooks. All procedures were approved by the Institutional Bioethics committee of the Regional Research Center “Dr. Hideyo Noguchi”, Universidad Autonoma de Yucatan. Householders from 5 houses per village were instructed to collect any triatomines present inside their houses, and were then visited every 3 months to take the triatomines to the laboratory. This method has been found to be highly reliable [9],[11],[13] and more sensitive than manual collections in the presence of limited colonization [14],[38]. Four houses from two of these villages (Dzidzilche and Eknakan) were sprayed with a standard dose of 50 mg/m^2^ of cyfluthrin in November 2000, and monitored every 2 weeks for up to 9 months to detect re-infestation using a combination of manual searches, mouse traps and household collections [27]. All field data were expressed as the average number of bugs collected/house-trimester with 95% confidence intervals.

### The population dynamics model

We modelled the dynamics of a non-domiciliated population of *T. dimidiata* by using the model of Gourbière et al. [16]. In this model, the egg and larval stages are pooled into a single immature stage, which is then divided into a number of sub-stages of equal duration corresponding to the time step of the model. The underlying assumption is that every individual spends a fixed time as an immature, and the outcome is that immature sub-stages are groups of age classes [39]. The matrix describing the demography of the vector within a house is a Leslie matrix, which we denote A. The model also includes a periodic immigration vector M to mimic the seasonal invasion of vectors observed in the Yucatan peninsula. The overall dynamical system can then be written:
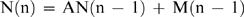
(1)where N(n) = (n_1_(n), n_2_(n), n_3_(n), n_A_(n)) included the number of females in three immature age classes and the number of adult females at the n^th^ time step and M(n) = (m_1_(n), m_2_(n), m_3_(n), m_A_(n)) the number of immigrants of the same categories (Note that we use index n instead of t as in Gourbière et al. [16] to refer to the main time step of the model, and t describes the smaller time-scale variations in the timing of insecticide spraying in this contribution (see below)). The time step of the model was fixed to 3 months to match model predictions with field data, which were determined every trimester, and to account for the average development time from egg to adult consistent with available data (see [16] for details). Accordingly, individuals of the first, second and third immature age classes are aged [0–90[, [90–180[ and [180–270] days, respectively. Because survival of individuals in these three immature age classes are considered identical, the Leslie matrix takes the form:
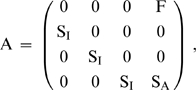
(2)where S_I_ and S_A_ are survival of immature and adults (probabilities per trimester), and F is female fecundity (female immature offspring per female-trimester). Because only adults immigrate into houses and because this only occurs between April and June [9], M(n) = (0,0,0,M) during the migration period, with M being the number of adult female immigrants, and M(n) = (0,0,0,0) during the remaining of the year.

Because the time unit desired to describe the control strategies in a flexible way is much shorter than the three-month time step previously selected, we adapted the above model to account for a daily description of the population dynamics, while keeping the three-month time step of the model. We divided each time step into T = 90 time units (t) and considered that immigrating individuals survive and reproduce proportionally to the time spent in the domestic habitat since their arrival at time τ. The population dynamics model is then divided into two parts, one describing the demography of individuals present in the domestic habitat since the beginning of the time step, and one accounting for the demography of individuals arriving at each time unit of the time step:
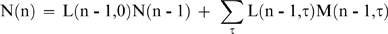
(3)L(n,τ) are Leslie matrices similar to L, but set up from survival S_I_(n,τ), S_A_(n,τ) and fecundity F(n,τ) defined over the time T-τ spent in the domestic habitat within the n^th^ time step. Similarly, M(n,τ) includes the number of immigrants at time τ of the n^th^ time step. We then used Equation 3 to simulate the vector population dynamics with or without control by changing the definition of parameters S_I_(n,τ), S_A_(n,τ), F(n,τ) and M(n,τ) according to the control strategies to be considered and the assumptions about their effects on vector demography. Finally, bug collection over the time steps was incorporated by removing a percentage p of individuals at the end of each day. The removed insects were summed over the duration of the time step to obtain a number of collected bugs/house-trimester, which is the model outcome that we compared to field observations. The best fits were obtained for p values 1–10%, with very limited changes in the quality of predictions over this range. For consistency, we thus display all our results for p = 5%.

### Fitting and testing the model with no control action

#### Modelling assumptions

While subdividing the time step into smaller time units, we assumed that within the time step 1) immature and adult survival are constant over time 2) adults immigrate at a constant rate, and 3) adults lay eggs regularly within the time interval left after they immigrated into the house. All the demographic parameters, S_I_(n,τ) S_A_(n,τ), F(n,τ) and M(n,τ), were specified according to those assumptions (See Protocol S1 for mathematical details).

#### Fitting the model

We estimated the demographic parameters by fitting the model with no control to field data from two villages during years 2000 and 2003. The data of both years and of all the immature stages were pooled to provide a reliable estimate of the yearly variations in vector abundance per house. The expected variations of the number of immature and adult individuals were calculated for a large range of values of each parameter. We calculated the sum of the square of the differences between observed and expected numbers of immatures and adults. The set of parameters providing the smallest sum of squares was retained and a Pearson correlation coefficient between observed and predicted bug abundance per house was used to measure the quality of the fit.

#### Testing the model

The ability of the model to predict bug abundance was measured by correlating the observed and predicted numbers of bugs. The test was performed on an independent data set coming from field studies carried out during years 2000, 2001 and 2003 in three villages different from those used to fit the model.

### Simulations of vector control interventions: Insecticide spraying

#### Modelling assumptions

Insecticide spraying was considered to reduce immature and adults survival according to the dose of insecticide present in the house. This effect on survival probabilities was described by a classical sigmoid dose-response relationship. The insecticide dose present was evaluated daily according to an exponential decay of the active ingredient starting on the day of application. In absence of quantitative data on the potential interaction between these two sources of mortalities, we assumed that they act independently and thus combine them multiplicatively to define the overall survival probability. Fecundity of adults was also decreased as a result of the impact of insecticide on immature and adult survival. All the demographic parameters, S_I_(n,τ), S_A_(n,τ) and F(n,τ), were then modified to account for insecticide spraying (See Protocol S1 for mathematical details).

#### Fitting the model

The model was fitted to field bug collections from a pilot insecticide trial performed in 2001 to estimate insecticide half-life (Table 1). Pearson correlation coefficient between observed and predicted bug abundance per house was used to measure the quality of the fit. The dose-response relationship (See Protocol S1) was established considering a LD_50_ = 32.2 mg/m^2^ and a LD_90_ = 182.4 mg/m^2^ (Table 1). These lethal doses derive from the experimental evaluation of the effect of cyfluthrin on *T. infestans* [40], and were considered similar to the effect of pyrethroids on *T. dimidiata* [41].

**Table 1:** Parameter values used to simulate vector population dynamics with and without control actions.

Parameter description	Estimate	Other tested values
Immature survival probability over 3 months (S_I_)	1(a)	0–0.01(c)
Adult survival probability over 3 months (S_A_)	0.224(a)	0.21(c)
Fecundity of females over 3 months (F)	0.434(a)	0.29(c)
Number of adult immigrating/year (M)	21.1(a)	1–25
Half-life of the insecticide in days (t_1/2_)	38(a)	15 days to 6 months [43]
50% lethal dose in mg/m^2^ (LD_50_)	32.2 [40]	15–100 mg/m^2^ [40],[45]
90% lethal dose in mg/m^2^ (LD_90_)	182.4 [40]	60–190 mg/m^2^ [40],[45]
Dose sprayed in mg/m^2^ (Q)	50(b)	10–250 mg/m^2^
Trimester of first spraying (n_fs_)	4(b)	1 to 4 (by 1)
Day of first spraying (t_ins_)	45(b)	0 to 60 (by 30)
Number of trimesters between two interventions (P)	none(b)	2 to 12 (by 2)
Reduction in immigration due to insect screens (r)	none	0 to 1 (by 0.1)
Reduction in survival and fecundity due to bednets (s)	none	0 to 1 (by 0.1)

(a) Estimated from the fit to field data.

(b) Values used to reproduce a unique spray on November 15^th^ as in the field trial [27] to estimate the half-life of the insecticide.

(c) Values estimated in Gourbière et al. [16].

#### Simulations of various strategies of spraying

For further simulation of interventions, we evaluated the effect of the spraying date of a single application by testing each month of the year (Table 1). We also tested single spraying of variable doses of insecticide as well as various spraying frequencies (Table 1). All these analyses were performed for the estimated half-life value, and we explored additional values ranging from 15 days to 6 months, according to estimates for various insecticides (Table 1). We also tested two additional dose-response relationships by varying LD_50_ and LD_90_ within a range of possible values (Table 1). Efficacy of control is expressed as percent reduction in bug number/house evaluated over a year when only one spray is applied, and over three years when repeated sprays are simulated.

#### Sensitivity analysis

Since the immigration rate has been shown to be the overwhelming factor in explaining non-domiciliated vector population dynamics, we varied this parameter from 1 to 25 immigrants per year according to estimates obtained from various methods [15],[16],[42]. We also performed a sensitivity analysis to the survival and reproductive abilities of individuals by repeating all the simulations described above using the demographic parameter estimates we previously obtained [16]. These parameter values (S_I_ = 0.01/trimester, S_A_ = 0.21/trimester, F = 0.29 female offspring/female-trimester) correspond to a sink population, with a growth rate equal to λ = 0.20. As expected, this sensitivity analysis resulted in quantitative changes in the abundance of insects. However, it did not alter any of our conclusions about the relative efficacy of the various strategies of spraying. We then present only the results obtained for the demographic parameter values estimated in this contribution (Table 1).

### Simulations of vector control interventions: Insect screens and bednets

#### Modelling assumptions

Door and window insect screens were considered as a physical barrier impeding the arrival of some of the immigrant vectors into the domestic habitat. Bednets were assumed to limit blood intake of the triatomines, leading to a decrease in survival and fecundity of the bugs. We thus modelled insect screens by multiplying the immigration M(n,τ) by a factor of bug exclusion r and bednets by weighting the fecundity F(n,τ) and survival S_I_(n,τ), S_A_(n,τ) by a factor of blood intake reduction s.

#### Simulations of various screens and bednets efficacy

Because no field data are available to estimate the reduction in triatomine immigration which may be expected by insect screens or the magnitude of the reduction of survival and fecundity due to bednets, we tested a complete range of reduction by varying r and s from 0 to 100%. The efficacy of control is expressed as percent reduction in bug number/house for one year following installation of screens or bednets.

#### Sensitivity analysis

We also varied the demographic rates as described above. Again, because there were only quantitative changes in the abundance of vectors, we present only the results obtained for the demographic parameter values estimated in this contribution (Table 1).

## Results

### Fit of the model to field data

**Figure 1:**
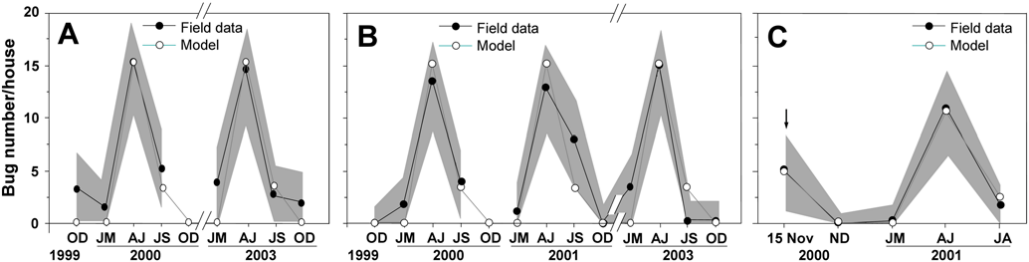
Fit and test of the model. (A) Fit of the model with no control actions. (B) Test of the predictive power of the fitted model. (C) Fit of the model with insecticide spraying. Field data are given with a 95% confidence interval (shaded area).

The model's demographic parameters were first fitted to two years of field data from two villages in the absence of vector control interventions. The optimal parameter values were M = 21.1 immigrants/year, S_I_ = 1/trimester, S_A_ = 0.434/trimester, F = 0.224 female offsprings/female-trimester, and these provided a very good fit of the model to field data for the total bug population (R^2^ = 0.953, Fig. 1A). This corresponded to a domestic population growth rate of λ = 0.83. In agreement with a previous estimate of λ = 0.20 obtained for another population [16], this confirmed that houses can truly be considered as sinks since λ<1 [19]. All the demographic parameter values were similar to those determined in our previous model [16], except for the survival of immatures. The unrealistically high value obtained is explained by the very low number of immatures in the population, resulting in a negligible weight to S in the overall quality of the fit. Using an immature survival probability of 0 only changed the least square value associated to the fit by 4.6%, whereas decreasing the amount of immigration to M = 1 lowered the quality of the fit by 2256%. This corroborated previous sensitivity analysis, where the effect of S_I_ was found to be 7 to 8 orders of magnitude lower than the effect of M (with Sobol standardized indices equal to 0.000005 and 0.89, respectively) [16]. We further tested the predictions of the model by comparing them with 3 years of independent field data from three other villages, which confirmed its very good predictive value to reproduce the observed seasonal variations in triatomine population (R^2^ = 0.891, Fig. 1B). All further calculations presented in this study were performed using demographic parameter values providing the best fit, but similar results were obtained when immature survival probability was forced to zero (data not shown). Insecticide spraying was then introduced into the model by reducing bug survival and fecundity values in a dose-dependent manner, and the model output was fitted to field data from a pilot trial to estimate insecticide half-life. The best fit of the model (R^2^ = 0.985, Fig. 1C) was obtained for a half-life of 38 days, which is in good agreement with the expected and measured half-life of pyrethroids and a lethal residual effect of about 3 months [40],[43],[44].

### Optimization of vector control with insecticide spraying

**Figure 2:**
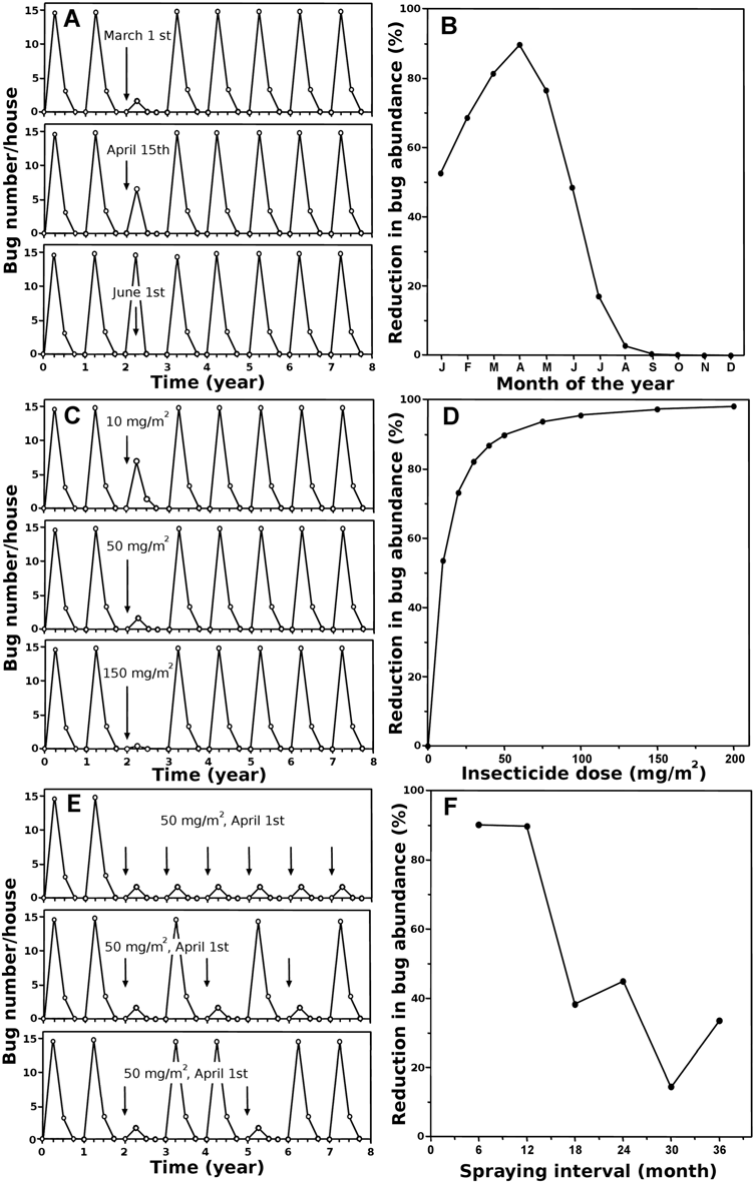
Optimization of insecticide spraying. (A–D) Single spray. (A) Variations in bug abundance. (B) Efficacy as a function of the date of spraying. (C) Variations in bug abundance with application of various insecticide dose. (D) Efficacy as a function of insecticide dose. (E,F) Repeated spraying. (E) Variations in bug abundance with repeated spraying. (F) Efficacy as a function of time interval between spraying.

Once we determined the model's parameters that best fitted field data, we predicted domestic bug abundance as a function of time after various control interventions. We first explored the effect of the timing of insecticide spraying during the year. The effects of a single insecticide spraying (50 mg/m^2^ at various dates) on bug abundance in the houses was only observed for a few months, and was followed by a rapid return to a normal cycle of infestation as soon as a new season of infestation occurred (Fig. 2A). Also, the timing of spraying during the year was critical for the magnitude of the reduction in bug abundance post-intervention (Fig. 2A and 2B). A maximum reduction in triatomine abundance of 90% for one year was achieved when spraying was conducted at the beginning of April, just before the start of the seasonal infestation. However, this maximum effect was only obtained for a very narrow time window, and efficacy dramatically decreased when spraying was applied before or after this period (Fig. 2B). Insecticide spraying had negligible effects (<5% reduction in bug abundance) when applied between August and December.

Although a standard cyfluthrin dose of 50 mg/m^2^ is commonly used for triatomine control [25], we evaluated the effect of varying this dose when the application is performed at the optimal time (April). A four-fold increase in insecticide dose (200 mg/m^2^) only provided a limited improvement in the reduction of bug abundance compared with the standard dose, and was not enough to sustain triatomine control for more than one seasonal infestation cycle (Fig. 2C and 2D). The standard dose of 50 mg/m^2^ thus provided a nearly optimal vector control. Nonetheless, an insecticide dose as low as 10 mg/m^2^ sprayed at the beginning of the infestation period was still able to reduce bug population by over 50% for a year (Fig. 2C and 2D).

Because a single insecticide spraying did not allow to achieve a sustainable vector control, we then evaluated the effects of repeated spraying and determined the optimal frequency of application. Our simulations clearly indicated that spraying once a year, just before the start of house invasion by adult triatomines, was the best strategy (Fig. 2E and 2F). Less frequent spraying led to a poor control during the seasons without insecticide application, whereas more frequent spraying did not increase the efficacy of the spraying.

**Figure 3:**
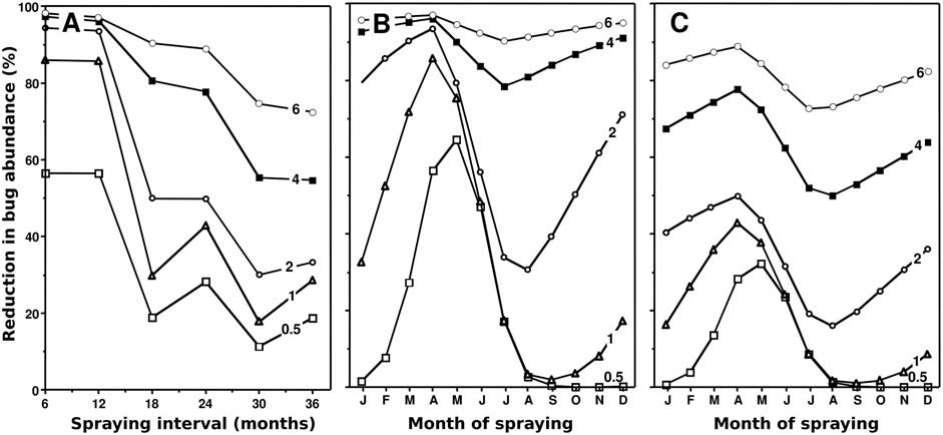
Effect of insecticide half-life. (A) Efficacy of repeated insecticide spraying as a function of the spraying interval and the insecticide half-life (indicated on each curve). (B) Efficacy of a yearly insecticide spraying. (C) Efficacy of spraying every two years.

Although our insecticide half-life estimate was in good agreement with expected values, we evaluated the robustness of the results using various half-life values in simulations where 50 mg/m^2^ are applied with various frequency at the optimal timing (April 1^st^). As expected, increasing insecticide half-life allowed for a more sustained vector control, leading to about 80% reduction in bug abundance by spraying every two years instead of one. However, a half-life of over 4 months was required for such a frequency of spraying to be effective (Fig. 3A). Similarly, the importance of the timing of insecticide application during the year decreased with longer half-life (Fig. 3B and 3C). Yearly interventions can be performed at any time when spraying insecticide with a half-life of over 4 months (Fig. 3B), but when spraying is conducted every two years, the timing of intervention still has to be considered even for insecticides with the highest half-life (Fig. 3C). Nonetheless, all our initial predictions remained valid for an insecticide half-life shorter than 2 months, for which the optimal strategy required yearly insecticide spraying during a narrow time window, just before the start of the seasonal house invasion by triatomines. These conclusions were valid for a wide range of LD_50_ of the insecticide, provided the spraying dose is adjusted accordingly, regardless of the level of immigration considered (Table 1, data not shown). Interestingly, the results of the above sensitivity analysis were found similar when considering the demographic parameter estimates from Gourbière et al., [16]. Our main conclusions on strategies of insecticide spraying thus hold for a wide range of non-domiciliated population dynamics because the two population growth rates tested, λ = 0.2 and λ = 0.83, cover most of the range of population growth rate corresponding to the definition of sink population, i.e., 0<λ<1.

### Evaluation of alternative vector control strategies

**Figure 4:**
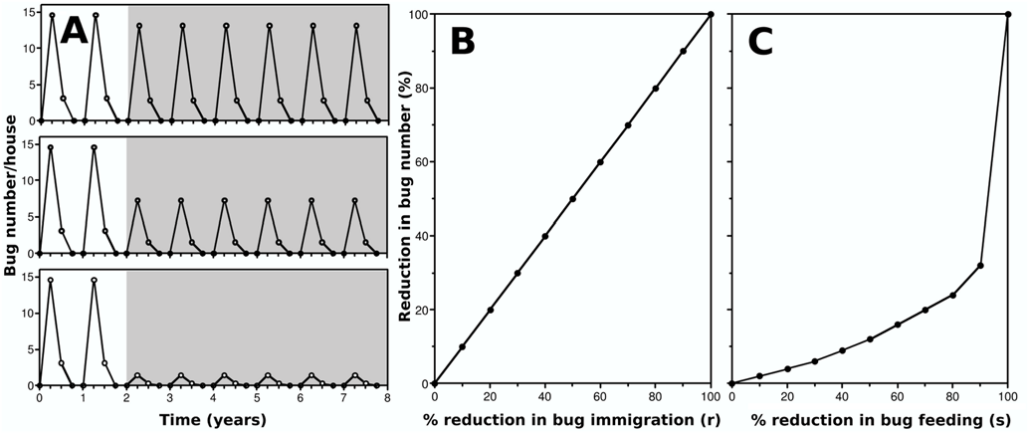
Evaluation of insect screens and bednets. (A) Variations in bug abundance with insect screens (gray shaded area) reducing bug immigration by 10 (top), 50 (middle), and 90% (bottom). (B,C) Efficacy of insect screens and bednets as a function of the percent reduction in bug immigration and bug feeding, respectively.

Given the importance of dispersal in triatomine population dynamics inside houses, we evaluated the effect of the presence of insect screens on doors and windows by reducing the immigration of bugs inside houses. Reduction in triatomine abundance in the houses was immediate following screens implementation, directly proportional to the reduction in bug immigration rate, and sustained for as long as the screens were maintained (Fig. 4A and 4B). We also simulated the use of non-impregnated bednets by considering that these reduced bug feeding, and thus bug survival and fecundity. While the effect of such bednets was sustained for as long as they were used, a reduction in bug survival and fecundity of up to 90% only accounted for a reduction in bug abundance of about 30% over a year. Smaller effects on survival and fecundity resulted in even smaller effects on bug abundance. The estimated efficacy of insect screens and bednets did not depend on the level of immigration considered and varied only slightly with the demographic parameters. (data not shown).

## Discussion

The integrative studies performed in the Yucatan peninsula provide a rare opportunity to develop mathematical models rooted in several years of field data. It was used here for the first time in an attempt at optimizing control strategies for non-domiciliated vectors of Chagas disease. The quality of the fit and of the predictive value of our deterministic model allowed to produce reliable simulations of a variety of control interventions. Also, while stochastic variations in the number of immigrants, which ultimately determine the number of individuals present in a given house, were not considered in our model, these are unlikely to qualitatively affect our results as indicated by our sensitivity analysis of immigrant numbers.

Simulations aimed at optimizing insecticide spraying clearly indicated that efficacy depended dramatically on the timing and frequency of spraying, both of which had to match closely the immigration season. This implies that a good understanding of the temporal pattern of immigration, which may differ between non-domiciliated triatomine species [6]–[8],[24],[38], is required for optimal control. On the other hand, variations in birth and death rates between individual genotypes or between species of vectors seems of little relevance to tune the optimal strategy of control for such sink populations. As long as the number of immigrant adult triatomines is controlled effectively, there remains virtually no individuals inside the houses after immigration, so that variations in the ability of these remaining insects to reproduce and survive inside the houses has only a minor impact on the percentage of reduction of their year-round abundance. In the case of *T. dimidiata* in the Yucatan peninsula and current pyrethroids, which have a half-life shorter than 2 months and have thus a residual lethal effect of about 1–6 months depending on the substrates [40],[43], a reduction of at least 80% in bug abundance would require yearly applications within a very short time window (March or April). While this may be feasible on a small scale, implementing such a control strategy on a large scale would require unrealistic logistics and a large cost of money. For example, based on a spraying capacity of 6–10 houses/day by a team of 2 people, spraying the ∼200,000 rural houses of the state of Yucatan in less than 2 months would require the simultaneous work of 400–650 teams during that time, together with a timely supply of insecticide in each village. Using an insecticide with a half-life >4 months would allow to either reduce spraying frequency to once every two years, or spray at any time of the year every year. It is interesting to note that the key factor for insecticide optimization against non-domiciliated triatomine is the half-life of the insecticide rather than its lethal effect or initial dose. This contrasts with the control of domiciliated triatomines, for which effectiveness of pyrethroids rests more on their initial impact rather than their residual effect [25]. Thus, while third-generation pyrethroids seem to be particularly adapted for the control of domiciliated triatomines [25], alternative insecticides with longer half-life such as fipronil [45], bifenthrin [44], or even the previously discarded organochlorines [25] may be more appropriate for the control of non-domiciliated bugs. However, their use may require strict management to avoid undesirable environmental and health impact, as well as the development of insecticide resistance, as already observed in some populations of triatomines [46]–[48].

Our results clearly indicate that none of these insecticide spraying interventions would be sustainable, since as soon as they are interrupted, re-infestation by new immigrant bugs occurs during the next season, implying large costs associated with repeated spraying. Some authors even suggested that control of non-domiciliated triatomines should not be considered, and that resources should rather be devoted to patient detection and care [2]. Nonetheless, alternative strategies may provide a more appropriate level of vector control.

Our simulations of insect screen effects indicate that an effective and sustained control can be achieved when a significant reduction of bug immigration is obtained. While it is difficult to estimate the possible efficacy of such screens in the field, an exclusion of over 90% of other insects has been observed with some greenhouse screens [49]. Also, a pilot field study of impregnated curtains used as a chemical barrier against non-domiciliated *R. robustus* resulted in a >60% reduction in live bugs collected over one month [30]. Our results are also consistent with the identification of such insect screens as a major protective factor against house infestation by *T. dimidiata* in urban Merida in the Yucatan [12]. A range of efficacy of insect screens of 70–90% would thus be very comparable to that of a yearly application of pyrethroids, but sustainable and hence less expensive. Even though our model did not take into account a decrease in efficacy of insect screens due to progressive tear-and-wear, it seems reasonable to consider that they would be effective for several years.

On the other hand, we found that bednets had little effect on bug abundance, possibly because triatomine reproductive output inside houses is already low in the absence of interventions [16],[18]. However, the potential of bednets cannot be ruled out from our results, since reduction in vector-human contacts, and thus parasite transmission, is not taken into account in our model, but has been observed in other settings [31],[50],[51]. Also, a number of additional vector control intervention have not directly been tested in this study, but their outcome can be predicted from our results. For example, insecticide-impregnated insect screens and bednets should reduce bug abundance, and their sustainability would depend on the half-life of the insecticide used for impregnation.

In conclusion, our study illustrates well the usefulness of coupling modelling and field studies to design and optimize effective control interventions and develop evidence-based public health policies, as previously done for the control of other diseases [33]–[35]. Our results clearly indicate that pyrethroid spraying is of limited usefulness for the control of non-domiciliated triatomines, while insect screens may be a simple, cost-effective and sustainable intervention. In addition, such screens would have an effect on all vector-borne diseases present, such as dengue, malaria or leishmaniasis [51],[52], and would thus be an excellent example of a high impact multi-disease intervention for the integrated control of neglected diseases [53]. Further field evaluations of the best vector control strategies identified here are warranted to confirm their efficacy and provide information on their implementation, including acceptability by the community and costs.

## Supporting Information

Alternative Language Abstract S1 Translation of the Abstract into Spanish by Eric Dumonteil
(0.07 MB PDF)

Alternative Language Abstract S2 Translation of the Author Summary into Spanish by Corentin Barbu
(0.07 MB PDF)

Alternative Language Abstract S3 Translation of the Author Summary into Portuguese by Sébastien Gourbière
(0.13 MB PDF)

Protocol S1 (0.33 MB PDF)
