# Supplementary material for: Optimization of Control Strategies for Non-Domiciliated Triatoma dimidiata, Chagas Disease Vector in the Yucatán Peninsula, Mexico
Source: PLoS Negl Trop Dis. 2009 Apr 14;3(4):e416. doi: 10.1371/journal.pntd.0000416 (PMC2664331; doi:10.1371/journal.pntd.0000416)
Supplement: Alternative Language Abstract S1 — Translation of the Abstract into Spanish by Eric Dumonteil (0.07 MB PDF) [file pntd.0000416.s001.pdf]

Resumen

Antecedentes. La enfermedad de Chagas es la enfermedad vectorial mas importante en América Latina. Iniciativas regionales basadas en la aplicación de insecticidas residuales han controlado exitosamente los vectores domesticados en varias regiones. Los vectores no-domiciliados son responsables de un riesgo de transmisión significativo y su control representa ahora un reto clave para el control de la enfermedad.

Metodología/Resultados principales. Desarrollamos un modelo matemático para predecir las variaciones temporales de la abundancia de vectores no-domiciliados en las casas. Los parámetros demográficos fueron estimados por el ajuste del modelo a dos años de datos de campo en la península de Yucatán, México. El valor predictivo del modelo fue evaluado utilizando un juego de datos independiente antes de examinar la eficacia de estrategias de control basadas en la aplicación de insecticida, mosquiteros y mosquiteros para cama. El modelo se ajustó con mucha precisión y predijo los datos de campo en ausencia y en presencia de una aplicación de insecticida. La aplicación de piretroide fue efectiva cuando se aplican 50 mg/m^2 anualmente y durante un periodo de dos meses que coincide con la estación de inmigración. La reducción de > 80% en la abundancia de insectos no mejora con dosis mas altas o mas frecuentes, y se reduce drásticamente si no esta sincronizada o con aplicaciones menos frecuentes. Alternativamente, el uso constante de mosquiteros reduce la abundancia de insectos, proporcionalmente a la reducción de la tasa de inmigración de los vectores.

Conclusión/Importancia. El control de los vectores no-domiciliados puede difícilmente lograrse con la aplicación de insecticidas, dado que se necesitaría una aplicación anual y un conocimiento detallado del patrón temporal de inmigración. Los

mosquiteros ofrecen una alternativa sustentable y efectiva, que puede ser parte de una intervención multi-enfermedad para el control integrado de enfermedades transmitidas por vectores.

Traducción del resumen de Eric Dumonteil
