# Supplementary material for: Optimization of Control Strategies for Non-Domiciliated Triatoma dimidiata, Chagas Disease Vector in the Yucatán Peninsula, Mexico
Source: PLoS Negl Trop Dis. 2009 Apr 14;3(4):e416. doi: 10.1371/journal.pntd.0000416 (PMC2664331; doi:10.1371/journal.pntd.0000416)
Supplement: Alternative Language Abstract S2 — Translation of the Author Summary into Spanish by Corentin Barbu (0.07 MB PDF) [file pntd.0000416.s002.pdf]

Resumen del author

La enfermedad de Chagas es la enfermedad de origen vectorial más importante en América Latina. La pulverización de insecticida residual ha sido utilizada exitosamente para la eliminación de vectores domésticos en muchas regiones. No obstante, algunos vectores que no son de origen doméstico son capaces de invadir hogares; éstos son actualmente el desafió para un mejor control de la enfermedad. Hemos desarrollado un modelo matemático para predecir las variaciones temporales de la abundancia de vectores no domiciliados al interior de hogares, basados en parámetros demográficos de los insectos. La fiabilidad de las predicciones se demuestra a través de la comparación de las últimas con los datos obtenidos de la colecta de insectos realizada en la Península de Yucatán, México. Así, hemos simulado las estrategias de control vectorial basadas en la pulverización de insecticida, mosquiteros y mosquiteros para camas, para evaluar su eficacia en la reducción de triatominos en los hogares. Una óptima reducción de insectos de al menos 80% puede ser obtenida por medio de la aplicación de insecticida sólo cuando las dosis de al menos 50 mg/m^2 son aplicadas cada año durante un periodo de dos meses que coincide con la invasión del insecto al hogar. Alternativamente, el uso constante de mosquiteros reduce la abundancia de insectos en los hogares y ofrece una alternativa sustentable. Estos mosquiteros serán parte de intervenciones novedosas para el control integrado de varias enfermedades vectoriales.

Traducción del resumen del autor de Corentin Barbu

Chagas disease is the most important vector-borne disease in Latin America. Residual insecticide spraying has been used successfully for the elimination of domestic vectors in many regions. However, some vectors of non-domestic origin are able to invade houses, and they are now a key challenge for further disease control. We developed a mathematical model to predict the temporal variations in abundance of non-domiciliated vectors inside houses, based on triatomine demographic parameters. The reliability of the predictions was demonstrated by comparing these with different sets of insect collection data from the Yucatan peninsula, Mexico. We then simulated vector control strategies based on insecticide spraying, insect screens and bednets, to evaluate their efficacy at reducing triatomine abundance in the houses. An optimum reduction in bug abundance by at least 80 % could be obtained by insecticide application only when doses of at least 50 mg/m^2 were applied every year within a two months period matching the house invasion season by bugs. Alternatively, the use of insect screens consistently reduced bug abundance in the houses and offers a sustainable alternative. Such screens may be part of novel interventions for the integrated control of various vector-borne diseases.
