# Supplementary material for: Optimization of Control Strategies for Non-Domiciliated Triatoma dimidiata, Chagas Disease Vector in the Yucatán Peninsula, Mexico
Source: PLoS Negl Trop Dis. 2009 Apr 14;3(4):e416. doi: 10.1371/journal.pntd.0000416 (PMC2664331; doi:10.1371/journal.pntd.0000416)
Supplement: Alternative Language Abstract S3 — Translation of the Author Summary into Portuguese by Sébastien Gourbière (0.13 MB PDF) [file pntd.0000416.s003.pdf]

Tradução de a resumo de autor por Sébastien Gourbière

A doença de Chagas é a doença transmitida por insectos vectores mais importante na América Latina. Pulverizações de insecticidas com efeito residual têm sido usadas com sucesso na eliminação de vectores domésticos em varias regiões. No entanto, alguns vectores não domésticos são capazes de invadir as habitações e representam neste momento um desafio importante para o controlo futuro da doença. Desenvolvemos um modelo matemático para prever as variações no tempo da quantidade de vectores não domésticos no interior das residências, baseado em parâmetros demográficos da triatomina. A fiabilidade das previsões foi demonstrada comparando-as a vários conjuntos de dados de colecções de insectos da península do Yucatan, México. Simulámos em seguida diferentes estratégias de controlo dos vectores baseadas em pulverizações de insecticida, mosquiteiros para janelas e mosquiteiros, com o objectivo de avaliar a sua eficácia na redução da quantidade de triatomina nas habitações. Uma redução óptima da quantidade de insectos de pelo menos 80% foi obtida através da aplicação de insecticida, mas apenas quando uma dose mínima de 50 mg/m^2 era aplicada anualmente nos 2 meses correspondentes ao período de invasão das habitações pelos insectos. O uso de mosquiteiros para janelas reduziu de forma significativa e durável a quantidade de insectos nas habitações e representa uma alternativa sustentável. Estes mosquiteiros para janelas poderão fazer parte de novas intervenções no âmbito de um controlo integrado de diversas doenças transmitidas por vectores.
