# Supplementary material for: Optimization of Control Strategies for Non-Domiciliated Triatoma dimidiata, Chagas Disease Vector in the Yucatán Peninsula, Mexico
Source: PLoS Negl Trop Dis. 2009 Apr 14;3(4):e416. doi: 10.1371/journal.pntd.0000416 (PMC2664331; doi:10.1371/journal.pntd.0000416)
Supplement: Protocol S1 — (0.33 MB PDF) [file pntd.0000416.s004.pdf]

Protocol S1

Our population dynamics model (Equation 3) is expressed as function of demographic parameters, $S_I(n,\tau)$, $S_A(n,\tau)$, $F(n,\tau)$ and $M(n,\tau)$, whose definition need to be specified according to the additional assumptions made when considering the model with no control intervention and when using the model to implement various control strategies. In this appendix, we provide the mathematical details of those specifications.

The population dynamics model with no control actions

Since we assumed a constant survival of immature and adult individuals within a time step, one can write:

$$S_I(n,\tau) = S_I^{\frac{T-\tau}{T}} \qquad \text{Equation 4a}$$

and

$$S_A(n,\tau) = S_A^{\frac{T-\tau}{T}} \qquad \text{Equation 4b}$$

Because we assumed that adults lay eggs regularly, the contribution of individuals immigrating at τ to the production of immature in the next time step equals:

$$F(n,\tau) = f\sum_{t=\tau}^{T-1} S_A^{\frac{t-\tau}{T}} S_I^{\frac{T-t}{T}} \qquad \text{Equation 5}$$

where f is the number of eggs per laying. As expected if all individuals are present at the beginning of the time step ($\tau = 0$) and lay all their eggs at this time, the previous Leslie matrix is recovered since $S_I(n,0) = S_I$ and $F(n,0) = f\,S_I$. The latter expression corresponds to adult

fecundity F, i.e., the product of fertility by the immature survival over the duration of the time step.

Finally, since we assumed a constant rate of immigration, M(n,τ)=(0,0,0,M/T) during the invasion season, and M(n,τ) = (0,0,0,0) during the remaining of the year.

The population dynamics model with insecticide spraying

Assuming that natural mortality and mortality due to insecticide acted multiplicatively, the survival probability can be written:

$$S_I(n,\tau) = S_I^{\frac{T-\tau}{T}} \cdot \prod_{t=\tau}^{T-1} s_{ins}(n,t) \qquad \text{Equation 6}$$

where s_{ins} (n,t) accounts for both the variations of the quantity of insecticide present in the house at any time t within the nth time step, and the dose-response relationship to describe the consequences of insecticide on vector demography. The same relationship holds for adult survival substituting S_A to S_I.

Fecundity of adults was also decreased as a result of the impact of insecticide on immature and adult survival. The fecundity of adults in the treated houses was then written as:

$$F(n,\tau) = f \sum_{t=\tau}^{T-1} S_A^{\frac{t-\tau}{T}} \cdot S_I^{\frac{T-\tau}{T}} \cdot \prod_{t=\tau}^{T-1} s_{ins}(n,t) \qquad \text{Equation 7}$$

Modelling insecticide spraying only required to evaluate the amount of insecticide present in the domestic habitat at any time, and to calculate the reduction in immature and adult survival probabilities.

Equation for the variations of the amount of insecticide in the house.

To calculate the amount of insecticide $q_{ins}(n,t)$ present in the house at time unit t of any time step n, we considered an exponential decay of the active ingredient and referred to $t_{1/2}$ as the

insecticide half-life. We allowed for control strategies (Q, P) corresponding to sprays of a quantity Q of insecticide every P time units. We denote n_{fs} the time step of the first spraying and t_{ins} the time unit at which the insecticide is sprayed within the time step. The quantity $q_{ins}(n,t)$ for any couple (n,t) > (n_{fs}, t_{ins}) can then be written as a sum of the residual quantity of insecticide since the first application. A straightforward calculation leads to:

$$q_{ins}(n,t) = Q\sum_{j=1}^{N} \exp\left(\frac{-\ln 2}{t_{1/2}}\left[\left(t-t_{ins}\right)+T\left(\left(n-n_{fs}\right)-\left(j-1\right)P\right)\right]\right) \qquad \text{Equation 8}$$

where $N = 1 + \text{int}\left[\frac{n-n_{fs}}{P}\right]$ is the total number of insecticide sprays.

Dose-response curve to describe the consequences of insecticide on vector demography.

To calculate the reduction in immature and adult survival we used a classical sigmoid dose-response described by a logistic equation which applied to $s_{ins}(n,t)$ the survival rate to insecticide for 24 hours gives:

$$s_{ins}(n,t) = 1 - \frac{1}{1+\left(\frac{LD_{50}}{q_i(n,t)}\right)^b} \qquad \text{Equation 9}$$

where LD_{50} is the dose that cause the death of 50 % of the population in 24h, and b is the hill slope of the sigmoid. b itself can be expressed with respect to LD_{50} and LD_{90}, the dose killing 90 % of the population in 24h, as follows:

$$b = \frac{\ln\left(\frac{1}{0.9}-1\right)}{\ln\left(\frac{LD_{50}}{LD_{90}}\right)} \qquad \text{Equation 10}$$
